# Emerging Roles of Sympathetic Nerves and Inflammation in Perivascular Adipose Tissue

**DOI:** 10.1007/s10557-019-06862-4

**Published:** 2019-02-12

**Authors:** Sophie N. Saxton, Sarah B. Withers, Anthony M. Heagerty

**Affiliations:** 1 Division of Cardiovascular Sciences, Manchester Academic Health Science Centre, University of Manchester, Core Technology Facility (3rd floor), 46 Grafton Street, M13 9NT Manchester, UK; 2 School of Environment and Life Sciences, University of Salford, Manchester, UK

**Keywords:** Adipose tissue, Inflammation, Obesity, Sympathetic nerves, Exercise

## Abstract

Perivascular adipose tissue (PVAT) is no longer recognised as simply a structural support for the vasculature, and we now know that PVAT releases vasoactive factors which modulate vascular function. Since the discovery of this function in 1991, PVAT research is rapidly growing and the importance of PVAT function in disease is becoming increasingly clear. Obesity is associated with a plethora of vascular conditions; therefore, the study of adipocytes and their effects on the vasculature is vital. PVAT contains an adrenergic system including nerves, adrenoceptors and transporters. In obesity, the autonomic nervous system is dysfunctional; therefore, sympathetic innervation of PVAT may be the key mechanistic link between increased adiposity and vascular disease. In addition, not all obese people develop vascular disease, but a common feature amongst those that do appears to be the inflammatory cell population in PVAT. This review will discuss what is known about sympathetic innervation of PVAT, and the links between nerve activation and inflammation in obesity. In addition, we will examine the therapeutic potential of exercise in sympathetic stimulation of adipose tissue.

## Introduction

Obesity is increasingly prevalent and is currently estimated to affect around 30% of adults in the UK [1]. Obesity is associated with a cluster of health conditions known as the metabolic syndrome, including hypertension and type-II diabetes, all of which may contribute to reduced life expectancy [2, 3]. It is estimated that obesity costs the UK’s National Health Service £4.2 billion per year [4], which presents a considerable burden to society. Therefore, there is a need to transform obese patients into metabolically fit individuals. One might assume that the simple solution is weight loss; however, clinical studies have indicated that most patients will regain weight lost through diet [5]. This highlights a need for new therapeutic strategies in place of caloric restriction.

We live in an age of convenience; high-calorie foods are readily available, with less need to expend energy. As a result of excess energy intake, adipose tissue depots expand. However, adipose tissue is not just an energy store and is actually a highly metabolically active endocrine organ [6–9]. Perivascular adipose tissue (PVAT) surrounds a number of peripheral vascular beds and is comprised of dynamic cell populations in addition to adipocytes, which includes nerves, stem cells, microvasculature and a plethora of immune cells [6, 10, 11]. There is a wealth of evidence that PVAT secretes vasoactive factors, which modulate vascular tone in isolated arteries [12–14]. Many of these factors are reported to have anti-contractile effects on resistance arteries, skeletal muscle arteries and the aorta, reducing peripheral resistance [11, 15–17]. Therefore, these factors are likely to contribute to modulating blood pressure in vivo [14]. In obesity, where autonomic dysfunction is known to occur, PVAT becomes inflamed and its secretory profile becomes dysfunctional [11, 18]. Loss of PVAT function may contribute to the vascular complications associated with obesity such as hypertension and type-II diabetes [19–22].

In this review, we will discuss the roles of sympathetic innervation in adipose tissue phenotype and function, and highlight the consequences of autonomic dysfunction within PVAT. In addition, we will cover the inflammatory environment in obese PVAT, and explore the links between sympathetic innervation and inflammation.

## Adipocytes: More than Fat Stores

There are two classical and functionally different types of adipocytes: white and brown. White adipose tissue (WAT) is widespread in the body, comprising the majority of visceral, subcutaneous and perivascular adipose depots, and one function is to provide support and insulation for the organs and vessels it surrounds [23]. White adipocytes are most widely recognised as a form of energy storage, and they contain a large lipid droplet for storing free fatty acids [24]. Therefore, it is white adipocytes which proliferate and expand in obesity. However, the belief that WAT is purely an energy store has long since passed, and we now know that adipocytes secrete almost 100 different proteins including vasoactive adipokines and inflammatory cytokines which play a number of roles including modulation of vascular tone, insulin resistance and body weight regulation [23, 25–27]. In addition to this diverse secretory profile, adipocytes express receptors for a number of substrates including neurotransmitters, indicating a role for the nervous system in regulating adipocyte function.

Whilst white adipocytes store energy, brown adipocytes expend it, and are well known for their role in thermogenesis, particularly in infants. These smaller adipocytes, with a large number of mitochondria, produce heat through uncoupled respiration [23, 28, 29]. Brown adipose tissue (BAT) depots are highly vascularised compared to WAT, which allows for efficient dispersal of heat. Since brown adipocytes are associated with energy expenditure, it has been proposed that brown adipocytes may confer metabolic improvements in obesity, where there is an imbalance between energy intake and expenditure [30, 31]. Indeed in mouse models, BAT transplants have demonstrated the potential usefulness of brown adipocytes. When BAT was transplanted into the visceral cavity of obese mice, insulin resistance was completely reversed and whole body metabolism increased, which reduced total body fat mass [32, 33]. However, whilst BAT is widespread in the mouse, BAT in humans mostly disappears between infancy and adulthood [34], although functional BAT is present in the region between the anterior neck and thorax [30]. The importance of these findings lies in the recently developed field of adipose tissue ‘beiging’. It is possible to stimulate white adipocytes to differentiate into a new adipocyte phenotype; the beige adipocyte, which bears resemblance to brown adipocytes in its number of mitochondria, and therefore, its ability to participate in thermogenesis [35, 36]. This process can be stimulated by increases in healthy sympathetic stimulation, i.e. exercise, as well as cold exposure and caloric restriction [35, 37–39]. The physiological importance of sympathetic nerve-induced beiging will be discussed below.

The composition of PVAT varies with its anatomical location [10, 11, 40–43. Mesenteric and aortic PVAT is the most widely studied. Mesenteric PVAT primarily consists of WAT, although its adipocytes are relatively smaller than other visceral adipose depots [44, 45]. Expression of lipolytic genes in mesenteric PVAT is high; therefore, the rate of both basal and catecholamine-induced lipolysis is high in this vascular bed [44, 46]. In addition, expression of vascular endothelial growth factor is high, which is vital for expansion of adipose tissues in obesity [47]. Around the thoracic aorta, PVAT consists of BAT, whereas the abdominal aortic PVAT is a mixture [26, 43, 48]. Interestingly, this change in composition is thought to be linked to the development of atherosclerosis; the abdominal aorta is most susceptible to atherosclerosis, and expression of inflammatory markers in abdominal aortic mixed PVAT is high [48]. As discussed above, the BAT in thoracic aortic PVAT could have a protective effect. There have been some studies on PVAT surrounding coronary arteries. These studies indicate that, like mesenteric PVAT, coronary PVAT consists of small white adipocytes [49]. However, the secretory profile of coronary adipocytes is different. Expression of the vasodilator adipokine, adiponectin is low, and expression of pro-inflammatory factors is high [50]. This has been linked to development of hypertension and myocardial infarction [51–53].

## Sympathetic Innervation of Adipose Tissue

The sympathetic nervous system (SNS) modulates lipolysis of WAT. Sympathetic denervation will impair lipolysis, and increase WAT expansion [54–57]. Conversely, SNS activation will increase lipolysis [58]. In addition, this increase in lipolysis can be reduced by β-adrenoceptor inhibition. These studies clearly indicate that adipocytes express adrenoceptors which will respond to sympathetic nerve-derived catecholamines.

Electrophysiology studies confirm that sympathetic nerves are present in both WAT and BAT [59, 60], although the degree of activity in response to certain stimuli does vary between the two types [61]. In particular, SNS activity in WAT is increased by glucoprivation, cold exposure and food deprivation, increasing lipolysis and release of free fatty acids (FFAs). Whereas in BAT, only cold exposure will increase nerve activity, resulting in increased thermogenesis. Studies using retrograde viral tract tracers have indicated that nerve fibres present in adipose tissue originate from the general sympathetic nerve outflow of the central nervous system [55, 62, 63]. These studies also indicated the presence of sensory nerve fibres. The function of these sensory nerve fibres and their interaction with sympathetic nerves is unclear. It is likely that the sensory nerves sense products of lipolysis, feeding back onto the SNS in order to regulate lipolysis [62, 64]. However, a recent study has indicated that sensory nerves may directly stimulate the release of leptin from PVAT, which has a vasorelaxant effect on the vasculature [65].

The tight packing of adipocytes has made it difficult to visualise direct contact between nerves and adipocytes, and histological studies are conflicting. Studies using markers for sympathetic nerves agree that they are present in both BAT and WAT depots, including visceral, subcutaneous and perivascular depots [19, 66–68]. Whilst most tissues receive dual autonomic innervation, i.e. sympathetic and parasympathetic, 97–98% of nerves in adipose tissue are sympathetic [69]. Interestingly, a marker for vascular specific sympathetic nerves, neuropeptide tyrosine, is negative in BAT, indicating that adipocytes are innervated by a separate nerve population to the vasculature [67]. Whilst some studies have indicated a high degree of direct contact between nerves and adipocytes [66, 70, 71], others have determined that only 2–3% of adipocytes are directly innervated [68]. Therefore, it remains to be confirmed if adipocytes are directly innervated, or are responding to catecholamine spill over.

We have recently confirmed that the anti-contractile effect of PVAT in vitro is dependent upon sympathetic nerves [19]. Electrical stimulation (activating sympathetic nerves) of small mesenteric resistance arteries with and without PVAT demonstrates that PVAT exerts an anti-contractile effect, which can be abolished using pharmacological sympathetic denervation of the adipose tissue. Similar effects have been reported in the superior mesenteric artery, whereas in the aorta electrical stimulation induces a pro-contractile effect [15]. The difference between the two vascular beds can be explained by the adipose tissue phenotype: mesenteric PVAT comprises WAT, whereas aortic PVAT is BAT. Interestingly, the anti-contractile effect of PVAT in the mesenteric bed is absent in the spontaneously hypertensive rat, indicating the importance of PVAT function in blood pressure regulation [15]. In addition, when a greater electrical stimulus is used, mesenteric PVAT will exert a pro-contractile effect on the vasculature [72], suggesting that increased nerve activity will alter PVAT function.

## PVAT Contains an Adrenergic System

As discussed previously, catecholamines induce lipolysis in WAT via adrenoceptors on adipocytes, in particular β_3_-adrenoceptors [71, 73]. In BAT, β_3_-adrenoceptors stimulate thermogenesis [74]. Previously, these metabolic functions were thought to be the only roles for adrenoceptors on adipocytes [75]; however, we now know that adipocyte β_3_-adrenoceptors regulate vascular tone. In larger conductive vessels, β_3_-adrenoceptors are present in the endothelium, and when stimulated will mediate vasorelaxation [76–78]. However, they are not present in the small resistance arteries which modulate blood pressure. Despite this, a specific β_3_-adrenoceptor agonist induces hypotension in canines and rodents [79]. Briones et al. [80] demonstrated that this same agonist will induce vasodilation in small arteries, only when PVAT is left intact. Similarly, the agonist induces PVAT-dependent hyperpolarisation of vascular smooth muscle cells [81]. Recently, using β_3_-adrenoceptor antagonists, we have confirmed that β_3_-adrenoceptors are vital in the PVAT anti-contractile effect, and that stimulation of β_3_-adrenoceptors triggers secretion of the vasodilator adiponectin from PVAT [19].

It is worth noting that all adrenoceptor subtypes are present on adipocytes, and they all play a role in modulating lipolysis (reviewed by Lafontan and Berlan [82]). Whilst β-adrenoceptors are stimulatory, α-adrenoceptors inhibit lipolysis. However, it appears that only adipocyte β_3_-adrenoceptor contribute to the PVAT anti-contractile effect.

The existence of a catecholamine system within adipocytes was first demonstrated by Pizzinat et al. [83]). This group reported the expression of monoamine oxidase A and B in adipocytes, indicating that adipocytes will uptake and metabolise monoamines such as noradrenaline (NA). Mesenteric WAT adipocytes [19, 84] and BAT [85] express organic cation transporter 3 (OCT3). OCT3 is a member of solute carrier family SLC2AA, and plays a vital role in extraneuronal transport of neurotransmitters such as NA into the periphery [86–88]. We have recently shown that OCT3 plays a functional role in PVAT anti-contractile effect, by enabling adipocytes to sequester sympathetic nerve-derived NA.

In addition to metabolising NA, it is possible that adipocytes are a source of NA [89]. When denervated aortic BAT and mesenteric WAT is stimulated with tyramine, the adipose tissue releases functional NA [89]. However, as discussed below, PVAT contains a diverse immune cell population, many of which can produce NA [90, 91]. Therefore, further investigation is required to determine if it is the adipocytes themselves producing NA.

## Autonomic Imbalance in Obesity

It is widely accepted that the SNS is pathologically overactive in obesity [92, 93]. Overactivity has been confirmed in multiple skeletal muscle studies [94–96], as well as studies of the cardiac and renal nerves [97]. Most importantly, the degree of SNS overactivity positively correlates with body fat mass, and varies with the location of increased adiposity. In particular, SNS activity is highest in patients with excessive visceral adipose tissue [98]. The causative mechanisms of SNS overactivity are not well understood (reviewed by Lemche et al. [99] and Smith and Minson [92]); however, it is likely due to an imbalance in the hypothalamic-pituitary axis [100]. Changes in adipokine secretion will contribute to this imbalance. Leptin in particular is greatly increased in obesity, and has a stimulatory effect on the SNS; therefore, it is highly likely that leptin may be one driver of SNS overactivity in obesity.

One consequence of SNS overactivity in obesity is impaired catecholamine-induced lipolysis, which will contribute to adipocyte hyperplasia and hypertrophy [101–104]. Basal lipolysis however, is increased [105]. This may be due to increases in expression of inflammatory cytokines, discussed further below, which exert a stimulatory effect on lipolysis [106, 107]. This increase in basal lipolysis will result in an increase in circulating FFAs, which will contribute to development of insulin resistance, glucose intolerance and hypertension through effects on skeletal muscle and the renin-angiotensin-aldosterone system [108–111]. Studies in first-degree relatives of obese patients indicate that catecholamine-induced lipolysis is impaired in the offspring of obese patients [112]. This may suggest that impaired lipolysis occurs before expansion of adipose tissues. Indeed some studies have indicated that SNS overactivity occurs alarmingly early in response to a high-fat diet, before significant weight gain. In rats, only 12 days of a cafeteria diet induces SNS overactivity [113]. Similar rapid changes in SNS activity have been reported in humans with small changes in body weight, and the magnitude of SNS overactivity increases with the amount of weight gained [114].

**Fig. 1:**
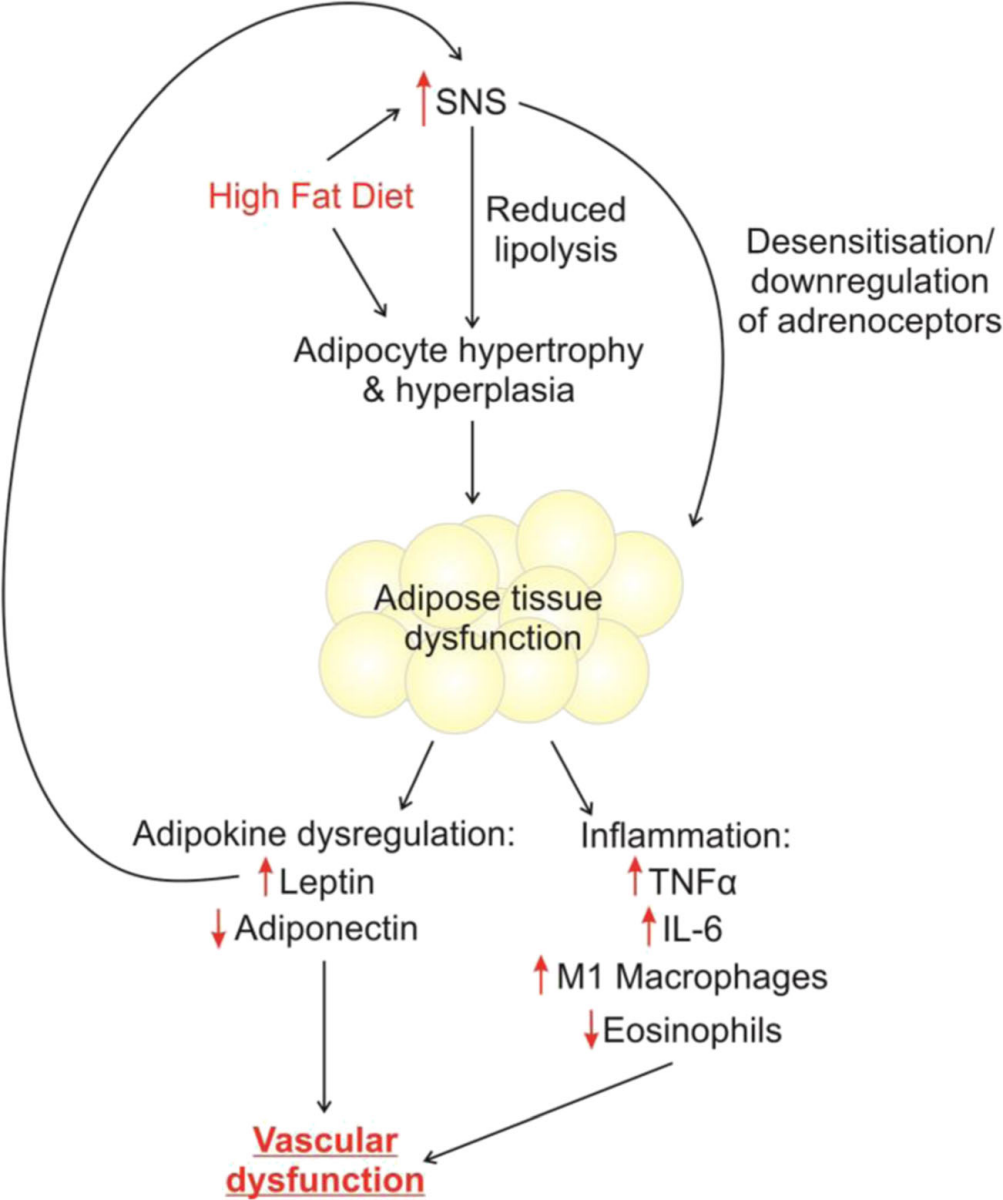
Consequences of SNS overactivity in PVAT. A high-fat diet will increase adipose tissue mass. In addition, in response to a high-fat diet, sympathetic nervous system (SNS) activity increased. One consequence of SNS overactivity is impaired catecholamine-induced lipolysis, which will contribute to increased adipose mass. It is possible that SNS overactivity may cause desensitisation and internalisation of adipocyte adrenoceptors. As a result of SNS overactivity, adipose tissue becomes inflamed and its secretory profile is altered. In particular, leptin secretion is increased, which will contribute to autonomic imbalance. Secretion of the vasodilator adiponectin is reduced, which will have direct effects on the vasculature, and may increase arterial tone. Infiltration of pro-inflammatory M1 macrophages is increased, which will increase expression of inflammatory cytokines including tumour necrosis factor α (TNF-α) and interleukin-6 (IL-6). Expression of anti-inflammatory cytokines is reduced. Changes to the immune cell populations will contribute to vascular dysfunction

With regard to PVAT function, it is possible that SNS overactivity will contribute to PVAT dysfunction in obesity. In heart failure patients, SNS overactivity results in desensitisation and internalisation of β-adrenoceptors [115]. It is possible that a similar effect occurs in adipose tissue, and SNS overactivity may result in β_3_-adrenoceptor internalisation in adipocytes, leading to reduced adiponectin release, and a loss of PVAT anti-contractile function. However, this has not yet been explored. For a summary of the potential effects of SNS overactivity in obesity, see Fig. 1.

## Obesity-Induced Inflammation of PVAT

Numerous models of obesity, including genetic [116, 117] and diet-induced [11, 118], demonstrate a loss of the PVAT anti-contractile effect, which likely contributes to development of hypertension. However, not all obese patients develop hypertension, and not all hypertensive patients are obese. A key common feature between obese hypertensives and lean hypertensives is the inflammatory populations in adipose tissue [119, 120]. PVAT contains a rich population of innate and adaptive immune cells [121], which is rapidly altered in obesity. In obesity, the increased need for energy storage results in adipocyte hypertrophy [11]. In addition, catecholamine-induced lipolysis is impaired [101]; this may be contributory to changes in catecholamine sensitivity which has been independently related to systolic blood pressure changes in a study of healthy, normotensive patients, and therefore, may have profound effects in hypertension [122]. Hypertrophy is not accompanied by increases in vascularisation, leading to hypoxia and subsequent oxidative stress and chronic inflammation [123, 124]. When subjected to hypoxia in vitro, the loss of PVAT function in obesity can be replicated in small resistance arteries from mice and rats [11, 18], indicating the importance of hypoxia-induced changes to the PVAT environment. Moreover, this loss of function can be reversed in vitro using antioxidants and cytokine antagonists [11, 18, 125]. One characteristic of hypoxia in obesity is an increase in expression of pro-inflammatory cytokines such as tumour necrosis factor alpha (TNF-α) and interleukin-6 (IL-6) [126, 127]. Application of antibodies for these cytokines can reverse PVAT dysfunction in hypoxia [11]. The role of TNF-α in hypertension is uncertain; some studies have shown that elevated TNF-α levels have been associated with the development of hypertension [128] whereas others using the DOCA-salt model of hypertension have shown no link [129]. This may indicate a differing role in different causes of hypertension. Further to this, there is strong evidence to support a role for IL-6 in hypertension both from experimental models [130] and human data [131].

Macrophages represent a large proportion of the immune cell population in PVAT, and in obesity macrophages are the main source of TNF-α and IL-6 [132]. Interestingly, loss of PVAT function in hypoxia can be prevented in the absence of macrophages [18], indicating the importance of macrophages in the inflammatory process of PVAT. In lean adipose tissue, the macrophage phenotype is predominantly M2 macrophages (alternatively activated macrophages) which release anti-inflammatory cytokines such as interleukin-10 [133], and are a source of catecholamines [90]. However, in obesity, there is a phenotypic switch from M2 macrophages to M1 macrophages (classically activated macrophages) [134]. In addition, macrophage infiltration is increased in both human and mouse obesity [18, 135]. Further to this, a ‘macrophage-centric model’ has been proposed to support the interrelatedness of neural, renal and vascular contributions to hypertension (reviewed by Harwani et al. [136],). These studies suggest that it is increased M1 macrophage activation and infiltration in obesity which contribute significantly to the increases in pro-inflammatory cytokine expression in obesity. It is worth noting that adipocyte-derived M1 macrophages may contribute directly to vascular dysfunction in obesity by reducing bioavailability of the vasodilator hydrogen sulphide in the endothelium [137], which has been known to develop prior to blood pressure changes in the spontaneously hypertensive rat [138].

Eosinophils are present in large numbers in healthy PVAT; however, levels are decreased in obesity [91]. Interestingly, the PVAT anti-contractile effect is lost in eosinophil-deficient mice, which is accompanied by increased blood pressure and reduced glucose tolerance. We have demonstrated previously that this phenotype and PVAT function can be rescued by eosinophil transplant [91], highlighting the importance of eosinophils in regulating PVAT function. Similar to M2 macrophages, eosinophils are a source of catecholamines, and are able to stimulate production of adiponectin and nitric oxide (NO) via activation of adipocyte β_3_-adrenoceptors [91]. Studies are emerging that parasitic induced eosinophilia may confer metabolic benefits in obesity. This hypothesis stems from the low incidence of cardiovascular disease in developing countries where helminth infections are prevalent [139, 140]. In mice, eosinophilia induced by a helminth infection demonstrates an increase in M2 macrophage activation, and has shown promise in improving glucose tolerance in obesity [141]. These effects may be mediated via interleukin-4 secretion from eosinophils, which has been shown to increase M2 polarisation [142]. IL-4 levels have been seen to be lower in hypertensive patients compared with control [143], and serum IL-4 and gene expression levels are reduced in patients with a particular cholesteryl ester transfer protein Taq1B polymorphism, and is associated with hypertension in this group [144]. However, the direct link between hypertension and eosinophils remains to be fully understood at this stage.

There are a number of other immune cells in PVAT which may contribute to pathogenesis in obesity, although their roles in adipose tissue are not well studied. Neutrophils are present at low levels in healthy PVAT, and their number is increased in obesity [145], and decreased following bariatric surgery [146]. Interestingly, neutrophil numbers correlate with blood pressure variability in lean hypertensive patients [147], indicating a role for neutrophils in vascular dysfunction. It is likely that their role in producing a variety of reactive oxygen species (ROS) is contributory to this observation [148]. Mast cells follow a similar pattern of expression in adipose tissue to neutrophils, and mast cell gene deficiency protects against diet-induced weight gain, and enhances glucose tolerance [149]. Mast cells are host to a panel of vasoconstrictor agents which are likely to underpin their contribution to vascular dysfunction. T and B cells are also increased in adipose tissue in obesity [150, 151], and both may be implicated in obesity pathogenesis. In a pharmacological model of hypertension in lean mice, T cell numbers are increased [152]. Data from lymphocyte-deficient *scid* mice implicated an eNOS- and COX-2-dependent pathway [153]. In diet-induced obese mice, treatment with B cell-depleting antibodies protects against insulin resistance and glucose intolerance [151]. All of these studies represent the importance of adipose tissue immune cell research in understanding vascular dysfunction in obesity.

It is important to note that expression of adiponectin, one of the potential adipokines responsible for the anti-contractile effect in health, has been shown to be reduced in hypoxia [154]. This decrease in adiponectin would likely worsen the inflammatory response, as adiponectin inhibits inflammatory cytokine production [155]. Additionally, adiponectin receptor 2 plays an important role in revascularisation following ischaemic injury [156]. Therefore, it is possible that reduced circulating adiponectin in obesity may prevent vascularisation of increased adipose depots, leading to hypoxia and inflammation.

## The Role of Sympathetic Nerves in Inflammation

The autonomic nervous system is integral to the inflammatory reflex [157]. The parasympathetic nerve-mediated cholinergic anti-inflammatory effect is well studied [158], whereas in comparison little attention is paid to the role of sympathetic nerves. Similar to parasympathetic nerves, sympathetic nerves have an important immunosuppressive role to play [159]. Whereas parasympathetic nerves regulate the number and function of lymphocytes, sympathetic nerves are involved in the control of granulocytes, including eosinophils, through adrenoceptors expressed on the surface of these cells [160]. Adrenoceptors are present on a number of immune cells, including splenocytes [161–163], macrophages [162, 164], T and B cells [165] and, as already mentioned, eosinophils [91]. Therefore, all of these immune cells will respond to sympathetic nerve-derived NA. Using retrograde tract tracers, sympathetic inputs to key components of the immune system including the thymus, bone marrow and lymph nodes have been well characterised [41, 166, 167]. Sympathetic input to lymph nodes is of particular significance in the context of this review, as PVAT is in close proximity to lymphatic organs, which likely enables the immune population in PVAT to alter rapidly in response to need [168]. In response to lipopolysaccharides, circulating TNF-α is increased, and this increase is greatly enhanced following sympathetic denervation of the spleen [163, 169]. Similarly, global sympathetic inhibition using reserpine elicited a similar enhancement of TNF-α production in response to lipopolysaccharides, and in this study, administration of β-adrenoceptor agonists reduced this enhancement of TNF-α production [162]. Macrophages are a large source of TNF-α, and express adrenoceptors; therefore, the effects of sympathetic denervation and reserpine on TNF-α may be mediated via increased macrophage activity. These studies indicate a clear role for sympathetic nerves in inflammation; therefore, it is likely that autonomic dysfunction in obesity may contribute to adipose inflammation (Fig. 1).

## Effects of Exercise on Autonomic Function and Inflammation

Exercise, which is considered to be a healthy, physiological form of sympathetic nerve activation, is well known to have beneficial effects in a number of diseases, including hypertension, diabetes and tachycardia [170, 171], and there is evidence that these beneficial outcomes may be mediated via effects on adrenoceptor activity, and on immune cells. In addition, exercise has been shown to reduce pathological sympathetic nerve activity occurring in obesity [172].

During exercise, the nutrient and oxygen demands of skeletal muscle is increased; therefore, there is a need for an acute increase in vasodilation to increase blood flow to the muscle via resistance arteries [173]. During this time, the contractility of vascular smooth muscle cells is altered [174, 175]. It is thought that this change in blood flow is controlled locally by chemical mediators produced by the muscle, red blood cells and endothelial cells [173, 176, 177]. However, the acute exercise-induced vasodilation is very rapid, and has been observed within 1 s of commencing exercise [178]. Therefore, it is likely that there is an initial contribution from the mechanical effect of muscle squeezing the vessels and pushing blood into the muscle [176, 179, 180]. This will be maintained after approximately 5 s by vasoactive chemicals. In addition, vascular tone is regulated by the autonomic nervous system, and during exercise, sympathetic nerve-mediated vasoconstriction is increased. This action of the nerves will limit vasodilation, preventing overloading of the heart with blood [181]. Interplay during exercise between sympathetic nerve evoked vasoconstriction, and chemically induced local vasodilation results in only moderate changes in blood pressure [173].

Chronic exercise is known to improve cardiovascular function; including increasing blood flow capacity [173], although, the molecular pathways and their links to the beneficial effects of exercise in disease are still being explored. It is likely that chronic exercise results in changes in gene expression and causes adaptations which will increase the efficiency of the vasculature in responding to the needs of muscle during exercise [182]. Chronic exercise training has been shown to increase flow-mediated dilation in large muscle arteries, and endothelium-dependent dilation in resistance arteries [183, 184]. Multiple studies have suggested that the increase in shear stress and stretching of arterial walls triggers altered gene expression in endothelial cells (reviewed by Whyte and Laughlin [173]). Indeed, some research groups have suggested that this is what should be considered the ‘normal’ phenotype in endothelial cells, and endothelial cells in sedentary individuals are a deviation from the norm [173, 177, 185]. Healthy endothelial function is vital in a number of processes, such as smooth muscle cell proliferation and migration, innate immunity and maintaining an anti-inflammatory environment (reviewed by Mensah [186]). Therefore, improvements in endothelial function using exercise could be useful in obesity.

There is a multitude of evidence that exercise alters adrenoceptor expression and sensitivity in cardiac tissue. In mice, exercise training protects against myocardial reperfusion injury via upregulation of β_3_-adrenoceptors [187]. Similarly, intense exercise in diabetic rats demonstrated increased β_3_-adrenoceptor expression in the heart [188], although a similar study using the same diabetes model, with a less extensive training protocol, indicated improvements in β_1_-adrenoceptor expression, and no effect on β_2/3_-adrenoceptor expression [189]. Likewise, dogs with ventricular fibrillation exhibited increased β_1_-adrenoceptor in cardiac tissue following exercise training [190]. The effect of exercise on adrenoceptor expression extends to immune cells. In healthy humans, immediately after one exercise session, β-adrenoceptor expression was increased in lymphocytes, although there were no studies to specify which isoform [191]. Most importantly, for the context of this review, in the Otsuka Long-Evans Tokushima Fatty rat, voluntary exercise on provided exercise wheels increased β_3_-adrenoceptor gene expression in adipose tissue [192].

Whilst the effects of exercise on adrenoceptor expression in the vasculature are not well studied, there is at least some evidence that exercise improves responsiveness of diseased arteries. In atherosclerotic pigs, exercise improved the response of coronary arteries to endothelin-1 [193]. In the obese Zucker rat, exercise improved the vasodilator response of skeletal muscle arteries to arachidonic acid, as well as improving glucose tolerance [194]. In human obese patients, exercise improved flow-mediated dilation in both the brachial artery and forearm microvasculature [195, 196].

Some studies have shown that exercise alters adipokine secretion in obesity. Adiponectin is increased following exercise [197], and leptin is decreased [197, 198]. In addition, TNF-α expression is reduced in the adipose tissue of exercised diet-induced obese mice [199]. A number of studies have reported similar effects of exercise on inflammatory cells in skeletal muscle. In diet-induced obesity models, forced exercise reduced TNF-α and T cell expression, macrophage infiltration and induced a phenotypic switch of M1 macrophages to M2 [200–202]. These findings were independent of weight loss. Similarly, in a voluntary exercise study, TNF-α expression was reduced, and this was accompanied by improvements in insulin sensitivity and glucose tolerance [199]. Interestingly, an acute exercise study involving only one swimming session reduced TNF-α in adipose tissue, increased M2 macrophage polarisation and improved glucose signalling [203].

Of course we cannot exclude the role of weight loss in the beneficial effects of exercise. Lipolysis of WAT is regulated by SNS, and as a result of increased energy expenditure during exercise, lipolysis is increased [204], which will reduce fat mass and therefore, body weight. Exercise does reduce adipocyte size, and will increase mitochondrial bioavailability [205–207], which fits with the increase in energy expenditure required by exercise. In addition, sympathetic activation by exercise is associated with reduced food intake [98].

## The Potential for Exercise-Induced Beiging of Adipose Tissue

As discussed previously, it is possible to stimulate white adipocytes to differentiate into beige adipocytes, which may confer the metabolic benefits of BAT. In a recent human trial, male obese patients were subjected to increasing periods of mild cold exposure (which is known to induce beiging [38, 39]) whilst their dietary intake was controlled [208]. Following only 10 days, patients exhibited significant improvements in glucose uptake, although there were no further studies to determine if this effect was sustained. The results of this study, and others like it, have made beiging adipocytes an attractive therapeutic target.

The activity of BAT is regulated by SNS [209], and as already discussed, exercise increases SNS activity. Therefore, it is likely that exercise will increase BAT activity. Many exercise studies, including running and swimming, have demonstrated that exercise stimulates beiging of WAT, and this is true for both forced and voluntary exercise [205, 210, 211]. Interestingly, the degree of beiging, quantified by measuring expression of uncoupling protein-1, does depend on the duration and type of exercise (reviewed by Dewal and Stanford [212]). Beiging appears to be much greater following periods of swimming than running, and most surprisingly, shorter durations of exercise (less than 3 weeks) are the most effective. It has been suggested that exercise-induced lipolysis, leading to a reduction in adipose mass [213], may become significant during longer training programmes.

Stanford et al. [211] have conducted a particularly illuminating study into the benefits of exercised-induced beiging. In this study, exercise wheels were provided to mice for voluntary exercise for 11 days. Using gene set analysis, Stanford et al. found that exercise increased expression of genes important to glucose metabolism as well as beiging. More excitingly, this group transplanted subcutaneous WAT from exercised trained mice into the visceral cavity of sedentary high-fat diet fed mice. Whilst the fat transplant had no effect on body weight, food intake or activity, 9 days post-transplant blood glucose, insulin and cholesterol were significantly improved in the high-fat diet mice. These effects were reduced 14 days post-surgery, and completely dissipated by 28 days. Nonetheless, these surprising results clearly demonstrate a beneficial effect of acutely exercised fat.

**Fig. 2:**
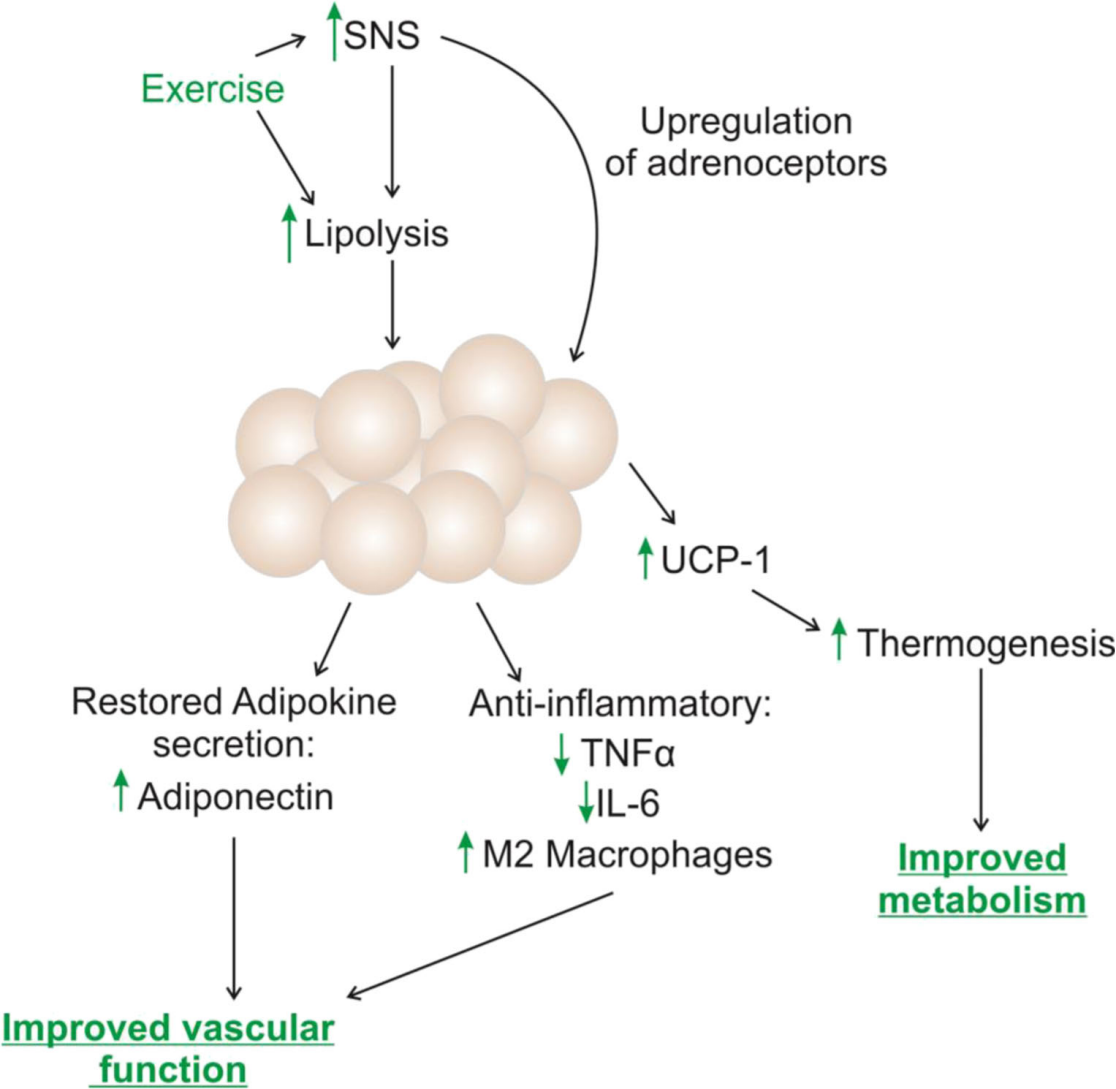
The potential benefits of exercise on obese PVAT. Exercise is considered healthy sympathetic nervous system (SNS) activity, and has been shown to have multiple benefits in obesity. Lipolysis is increased, contributing to weight loss, and exercise has been shown to improve adrenoceptor expression in cardiac tissue. In adipose tissue, the adipokine secretion profile is improved which will have direct effects on the vasculature. In addition, exercise reduces inflammation; tumour necrosis factor α (TNF-α) and interleukin-6 (IL-6) expression is reduced, and macrophages undergo a phenotypic switch to anti-inflammatory M2 macrophages. Exercise has been shown to induce beiging of white adipose tissue, quantified by expression of uncoupling protein-1 (UCP-1). This process will enable thermogenesis, which result in an improved metabolic profile via increased energy expenditure

A number of mechanisms behind exercise-induced beiging and the beneficial metabolic effects have been proposed which involve increased SNS activity. The simplest theory is that exercise increases BAT activity, which results in increased energy expenditure and therefore, weight loss [214]. Another suggestion is that exercise, or other factors which induce beiging, may disrupt fur growth, resulting in a need to increase thermogenesis and therefore, BAT. However, as discussed above, exercise appears to affect adrenoceptor expression and activity, and some studies have demonstrated that β_3_-adrenoceptor agonists induce beiging [215–217]. Similarly, the herbal supplement curcumin has been shown to stimulate beiging via increasing β_3_-adrenoceptor expression in WAT [218], and cold-induced beiging is dependent upon β_3_-adrenoceptors [219]. Therefore, it is likely that the exercised-induced beiging mechanism involves β_3_-adrenoceptors. As previously discussed, the PVAT anti-contractile effect is dependent upon β_3_-adrenoceptors; therefore, it is possible that exercise may be useful in restoring PVAT function in obesity via effects on β_3_-adrenoceptor expression and activity. For a summary of the potential effects of SNS activation using exercise in obesity, see Fig. 2.

## Summary

In addition to modulating metabolism, sympathetic nerves in PVAT modulate the release of vasoactive factors from adipocytes, which exert an anti-contractile effect on the vasculature; reducing peripheral resistance and therefore, blood pressure. Autonomic dysfunction in response to a high-fat diet contributes to adipose tissue expansion and recruitment of inflammatory cells in PVAT. Hypoxia and chronic inflammation result in PVAT dysfunction, and the overactivity of sympathetic nerves may be causing internalisation of adipocyte adrenoceptors. Healthy sympathetic hyperstimulation by exercise reduces expression of inflammatory cells in PVAT, and may improve expression of adrenoceptors; therefore, exercise may be useful in restoring PVAT function in obesity. In addition, exercise may be inducing transformation of white adipocytes to beige adipocytes, which confer an improved metabolic phenotype. Clearly, the study of sympathetic innervation of adipose tissue is vital to gain new insights into vascular dysfunction in obesity.

## Abbreviations

BAT: Brown adipose tissue
FFAs: Free fatty acids
IL-6: Interleukin-6
NA: Noradrenaline
OCT3: Organic cation transporter 3
PVAT: Perivascular adipose tissue
SNS: Sympathetic nervous system
TNF-α: Tumour necrosis factor α
WAT: White adipose tissue
